# Supplementary material for: Resolving the full spectrum of human genome variation using Linked-Reads
Source: Genome Res. 2019 Apr;29(4):635–45. doi: 10.1101/gr.234443.118 (PMC6442396; doi:10.1101/gr.234443.118)
Supplement: Supplemental Material [file supp_gr.234443.118_Supplemental_longranger-2.2.zip › longranger-2.2.tar.bz2!/lr2.2-public/lib/stan/cmdstan/stan/doc/api/html/skew__normal_8hpp_source.html]

Stan: src/stan/prob/distributions/univariate/continuous/skew\_normal.hpp Source File


|  |  |
| --- | --- |
|  | Stan  2.5.0  probability, sampling & optimization |


- Main Page
- Related Pages
- Namespaces
- Classes
- Files

- File List
- File Members

All Classes Namespaces Files Functions Variables Typedefs Enumerations Enumerator Friends Macros Pages

- src
- stan
- prob
- distributions
- univariate
- continuous

skew\_normal.hpp

Go to the documentation of this file.

1 #ifndef STAN\_\_PROB\_\_DISTRIBUTIONS\_\_UNIVARIATE\_\_CONTINUOUS\_\_SKEW\_\_NORMAL\_\_HPP

2 #define STAN\_\_PROB\_\_DISTRIBUTIONS\_\_UNIVARIATE\_\_CONTINUOUS\_\_SKEW\_\_NORMAL\_\_HPP

3

4 #include <boost/random/variate\_generator.hpp>

5 #include <boost/math/distributions.hpp>

6 #include <stan/prob/distributions/univariate/continuous/uniform.hpp>

7

8 #include <stan/agrad/partials\_vari.hpp>

9 #include <stan/math/error\_handling.hpp>

10 #include <stan/math/functions/owens\_t.hpp>

11 #include <stan/meta/traits.hpp>

12 #include <stan/prob/constants.hpp>

13 #include <stan/prob/traits.hpp>

14 #include <stan/math/functions/value\_of.hpp>

15

16 namespace stan {

17

18  namespace prob {

19

20  template <bool propto,

21  typename T\_y, typename T\_loc, typename T\_scale, typename T\_shape>

22  typename return\_type<T\_y,T\_loc,T\_scale,T\_shape>::type

23  skew\_normal\_log(const T\_y& y, const T\_loc& mu, const T\_scale& sigma,

24  const T\_shape& alpha) {

25  static const char\* function = "stan::prob::skew\_normal\_log(%1%)";

26

27  using std::log;

28  using stan::is\_constant\_struct;

29  using stan::math::check\_positive;

30  using stan::math::check\_finite;

31  using stan::math::check\_not\_nan;

32  using stan::math::check\_consistent\_sizes;

33  using stan::math::value\_of;

34  using stan::prob::include\_summand;

35

36  // check if any vectors are zero length

37  if (!(stan::length(y)

38  && stan::length(mu)

39  && stan::length(sigma)

40  && stan::length(alpha)))

41  return 0.0;

42

43  // set up return value accumulator

44  double logp(0.0);

45

46  // validate args (here done over var, which should be OK)

47  check\_not\_nan(function, y, "Random variable", &logp);

48  check\_finite(function, mu, "Location parameter", &logp);

49  check\_finite(function, alpha, "Shape parameter", &logp);

50  check\_positive(function, sigma, "Scale parameter", &logp);

51  check\_consistent\_sizes(function,

52  y,mu,sigma,alpha,

53  "Random variable","Location parameter",

54  "Scale parameter", "Shape paramter",

55  &logp);

56

57  // check if no variables are involved and prop-to

58  if (!include\_summand<propto,T\_y,T\_loc,T\_scale,T\_shape>::value)

59  return 0.0;

60

61  // set up template expressions wrapping scalars into vector views

62  agrad::OperandsAndPartials<T\_y, T\_loc, T\_scale, T\_shape>

63  operands\_and\_partials(y, mu, sigma, alpha);

64

65  VectorView<const T\_y> y\_vec(y);

66  VectorView<const T\_loc> mu\_vec(mu);

67  VectorView<const T\_scale> sigma\_vec(sigma);

68  VectorView<const T\_shape> alpha\_vec(alpha);

69  size\_t N = max\_size(y, mu, sigma, alpha);

70

71  DoubleVectorView<true,is\_vector<T\_scale>::value> inv\_sigma(length(sigma));

72  DoubleVectorView<include\_summand<propto,T\_scale>::value,

73  is\_vector<T\_scale>::value> log\_sigma(length(sigma));

74  for (size\_t i = 0; i < length(sigma); i++) {

75  inv\_sigma[i] = 1.0 / value\_of(sigma\_vec[i]);

76  if (include\_summand<propto,T\_scale>::value)

77  log\_sigma[i] = log(value\_of(sigma\_vec[i]));

78  }

79

80  for (size\_t n = 0; n < N; n++) {

81  // pull out values of arguments

82  const double y\_dbl = value\_of(y\_vec[n]);

83  const double mu\_dbl = value\_of(mu\_vec[n]);

84  const double sigma\_dbl = value\_of(sigma\_vec[n]);

85  const double alpha\_dbl = value\_of(alpha\_vec[n]);

86

87  // reusable subexpression values

88  const double y\_minus\_mu\_over\_sigma

89  = (y\_dbl - mu\_dbl) \* inv\_sigma[n];

90  const double pi\_dbl = boost::math::constants::pi<double>();

91

92  // log probability

93  if (include\_summand<propto>::value)

94  logp -= 0.5 \* log(2.0 \* pi\_dbl);

95  if (include\_summand<propto, T\_scale>::value)

96  logp -= log(sigma\_dbl);

97  if (include\_summand<propto,T\_y, T\_loc, T\_scale>::value)

98  logp -= y\_minus\_mu\_over\_sigma \* y\_minus\_mu\_over\_sigma / 2.0;

99  if (include\_summand<propto,T\_y,T\_loc,T\_scale,T\_shape>::value)

100  logp += log(boost::math::erfc(-alpha\_dbl \* y\_minus\_mu\_over\_sigma

101  / std::sqrt(2.0)));

102

103  // gradients

104  double deriv\_logerf

105  = 2.0 / std::sqrt(pi\_dbl)

106  \* exp(-alpha\_dbl \* y\_minus\_mu\_over\_sigma / std::sqrt(2.0)

107  \* alpha\_dbl \* y\_minus\_mu\_over\_sigma / std::sqrt(2.0))

108  / (1 + boost::math::erf(alpha\_dbl \* y\_minus\_mu\_over\_sigma

109  / std::sqrt(2.0)));

110  if (!is\_constant\_struct<T\_y>::value)

111  operands\_and\_partials.d\_x1[n]

112  += -y\_minus\_mu\_over\_sigma / sigma\_dbl

113  + deriv\_logerf \* alpha\_dbl / (sigma\_dbl \* std::sqrt(2.0)) ;

114  if (!is\_constant\_struct<T\_loc>::value)

115  operands\_and\_partials.d\_x2[n]

116  += y\_minus\_mu\_over\_sigma / sigma\_dbl

117  + deriv\_logerf \* -alpha\_dbl / (sigma\_dbl \* std::sqrt(2.0));

118  if (!is\_constant\_struct<T\_scale>::value)

119  operands\_and\_partials.d\_x3[n]

120  += -1.0 / sigma\_dbl

121  + y\_minus\_mu\_over\_sigma \* y\_minus\_mu\_over\_sigma / sigma\_dbl

122  - deriv\_logerf \* y\_minus\_mu\_over\_sigma \* alpha\_dbl

123  / (sigma\_dbl \* std::sqrt(2.0));

124  if (!is\_constant\_struct<T\_shape>::value)

125  operands\_and\_partials.d\_x4[n]

126  += deriv\_logerf \* y\_minus\_mu\_over\_sigma / std::sqrt(2.0);

127  }

128  return operands\_and\_partials.to\_var(logp);

129  }

130

131  template <typename T\_y, typename T\_loc, typename T\_scale, typename T\_shape>

132  inline

133  typename return\_type<T\_y,T\_loc,T\_scale, T\_shape>::type

134  skew\_normal\_log(const T\_y& y, const T\_loc& mu, const T\_scale& sigma,

135  const T\_shape& alpha) {

136  return skew\_normal\_log<false>(y,mu,sigma,alpha);

137  }

138

139  template <typename T\_y, typename T\_loc, typename T\_scale, typename T\_shape>

140  typename return\_type<T\_y,T\_loc,T\_scale,T\_shape>::type

141  skew\_normal\_cdf(const T\_y& y, const T\_loc& mu, const T\_scale& sigma,

142  const T\_shape& alpha) {

143  static const char\* function = "stan::prob::skew\_normal\_cdf(%1%)";

144

145  using stan::math::check\_positive;

146  using stan::math::check\_finite;

147  using stan::math::check\_not\_nan;

148  using stan::math::check\_consistent\_sizes;

149  using stan::math::owens\_t;

150  using stan::math::value\_of;

151

152  double cdf(1.0);

153

154  // check if any vectors are zero length

155  if (!(stan::length(y)

156  && stan::length(mu)

157  && stan::length(sigma)

158  && stan::length(alpha)))

159  return cdf;

160

161  check\_not\_nan(function, y, "Random variable", &cdf);

162  check\_finite(function, mu, "Location parameter", &cdf);

163  check\_not\_nan(function, sigma, "Scale parameter", &cdf);

164  check\_positive(function, sigma, "Scale parameter", &cdf);

165  check\_finite(function, alpha, "Shape parameter", &cdf);

166  check\_not\_nan(function, alpha, "Shape parameter", &cdf);

167  check\_consistent\_sizes(function,

168  y,mu,sigma,alpha,

169  "Random variable","Location parameter",

170  "Scale parameter","Shape paramter",

171  &cdf);

172

173  agrad::OperandsAndPartials<T\_y, T\_loc, T\_scale, T\_shape>

174  operands\_and\_partials(y, mu, sigma, alpha);

175

176  using stan::math::SQRT\_2;

177  using stan::math::pi;

178

179  VectorView<const T\_y> y\_vec(y);

180  VectorView<const T\_loc> mu\_vec(mu);

181  VectorView<const T\_scale> sigma\_vec(sigma);

182  VectorView<const T\_shape> alpha\_vec(alpha);

183  size\_t N = max\_size(y, mu, sigma, alpha);

184  const double SQRT\_TWO\_OVER\_PI = std::sqrt(2.0 / stan::math::pi());

185

186  for (size\_t n = 0; n < N; n++) {

187  const double y\_dbl = value\_of(y\_vec[n]);

188  const double mu\_dbl = value\_of(mu\_vec[n]);

189  const double sigma\_dbl = value\_of(sigma\_vec[n]);

190  const double alpha\_dbl = value\_of(alpha\_vec[n]);

191  const double alpha\_dbl\_sq = alpha\_dbl \* alpha\_dbl;

192  const double diff = (y\_dbl - mu\_dbl) / sigma\_dbl;

193  const double diff\_sq = diff \* diff;

194  const double scaled\_diff = diff / SQRT\_2;

195  const double scaled\_diff\_sq = diff\_sq \* 0.5;

196  const double cdf\_ = 0.5 \* erfc(-scaled\_diff) - 2 \* owens\_t(diff,

197  alpha\_dbl);

198  //cdf

199  cdf \*= cdf\_;

200

201  //gradients

202  const double deriv\_erfc = SQRT\_TWO\_OVER\_PI \* 0.5 \* exp(-scaled\_diff\_sq)

203  / sigma\_dbl;

204  const double deriv\_owens = erf(alpha\_dbl \* scaled\_diff)

205  \* exp(-scaled\_diff\_sq) / SQRT\_TWO\_OVER\_PI / (-2.0 \* pi()) / sigma\_dbl;

206  const double rep\_deriv = (-2.0 \* deriv\_owens + deriv\_erfc) / cdf\_;

207

208  if (!is\_constant\_struct<T\_y>::value)

209  operands\_and\_partials.d\_x1[n] += rep\_deriv;

210  if (!is\_constant\_struct<T\_loc>::value)

211  operands\_and\_partials.d\_x2[n] -= rep\_deriv;

212  if (!is\_constant\_struct<T\_scale>::value)

213  operands\_and\_partials.d\_x3[n] -= rep\_deriv \* diff;

214  if (!is\_constant\_struct<T\_shape>::value)

215  operands\_and\_partials.d\_x4[n] += -2.0 \* exp(-0.5 \* diff\_sq \* (1.0

216  + alpha\_dbl\_sq)) / ((1 + alpha\_dbl\_sq) \* 2.0 \* pi()) / cdf\_;

217  }

218

219  if (!is\_constant\_struct<T\_y>::value)

220  for (size\_t n = 0; n < stan::length(y); ++n)

221  operands\_and\_partials.d\_x1[n] \*= cdf;

222  if (!is\_constant\_struct<T\_loc>::value)

223  for (size\_t n = 0; n < stan::length(mu); ++n)

224  operands\_and\_partials.d\_x2[n] \*= cdf;

225  if (!is\_constant\_struct<T\_scale>::value)

226  for (size\_t n = 0; n < stan::length(sigma); ++n)

227  operands\_and\_partials.d\_x3[n] \*= cdf;

228  if (!is\_constant\_struct<T\_shape>::value)

229  for (size\_t n = 0; n < stan::length(alpha); ++n)

230  operands\_and\_partials.d\_x4[n] \*= cdf;

231

232  return operands\_and\_partials.to\_var(cdf);

233  }

234

235  template <typename T\_y, typename T\_loc, typename T\_scale, typename T\_shape>

236  typename return\_type<T\_y,T\_loc,T\_scale,T\_shape>::type

237  skew\_normal\_cdf\_log(const T\_y& y, const T\_loc& mu, const T\_scale& sigma,

238  const T\_shape& alpha) {

239  static const char\* function = "stan::prob::skew\_normal\_cdf\_log(%1%)";

240

241  using stan::math::check\_positive;

242  using stan::math::check\_finite;

243  using stan::math::check\_not\_nan;

244  using stan::math::value\_of;

245  using stan::math::check\_consistent\_sizes;

246  using stan::math::owens\_t;

247

248  double cdf\_log(0.0);

249

250  // check if any vectors are zero length

251  if (!(stan::length(y)

252  && stan::length(mu)

253  && stan::length(sigma)

254  && stan::length(alpha)))

255  return cdf\_log;

256

257  check\_not\_nan(function, y, "Random variable", &cdf\_log);

258  check\_finite(function, mu, "Location parameter", &cdf\_log);

259  check\_not\_nan(function, sigma, "Scale parameter", &cdf\_log);

260  check\_positive(function, sigma, "Scale parameter", &cdf\_log);

261  check\_finite(function, alpha, "Shape parameter", &cdf\_log);

262  check\_not\_nan(function, alpha, "Shape parameter", &cdf\_log);

263  check\_consistent\_sizes(function,

264  y,mu,sigma,alpha,

265  "Random variable","Location parameter",

266  "Scale parameter","Shape paramter",

267  &cdf\_log);

268

269  agrad::OperandsAndPartials<T\_y, T\_loc, T\_scale, T\_shape>

270  operands\_and\_partials(y, mu, sigma, alpha);

271

272  using stan::math::SQRT\_2;

273  using stan::math::pi;

274

275  VectorView<const T\_y> y\_vec(y);

276  VectorView<const T\_loc> mu\_vec(mu);

277  VectorView<const T\_scale> sigma\_vec(sigma);

278  VectorView<const T\_shape> alpha\_vec(alpha);

279  size\_t N = max\_size(y, mu, sigma, alpha);

280  const double SQRT\_TWO\_OVER\_PI = std::sqrt(2.0 / stan::math::pi());

281

282  for (size\_t n = 0; n < N; n++) {

283  const double y\_dbl = value\_of(y\_vec[n]);

284  const double mu\_dbl = value\_of(mu\_vec[n]);

285  const double sigma\_dbl = value\_of(sigma\_vec[n]);

286  const double alpha\_dbl = value\_of(alpha\_vec[n]);

287  const double alpha\_dbl\_sq = alpha\_dbl \* alpha\_dbl;

288  const double diff = (y\_dbl - mu\_dbl) / sigma\_dbl;

289  const double diff\_sq = diff \* diff;

290  const double scaled\_diff = diff / SQRT\_2;

291  const double scaled\_diff\_sq = diff\_sq \* 0.5;

292  const double cdf\_log\_ = 0.5 \* erfc(-scaled\_diff) - 2 \* owens\_t(diff,

293  alpha\_dbl);

294  //cdf\_log

295  cdf\_log += log(cdf\_log\_);

296

297  //gradients

298  const double deriv\_erfc = SQRT\_TWO\_OVER\_PI \* 0.5 \* exp(-scaled\_diff\_sq)

299  / sigma\_dbl;

300  const double deriv\_owens = erf(alpha\_dbl \* scaled\_diff)

301  \* exp(-scaled\_diff\_sq) / SQRT\_TWO\_OVER\_PI / (-2.0 \* pi()) / sigma\_dbl;

302  const double rep\_deriv = (-2.0 \* deriv\_owens + deriv\_erfc) / cdf\_log\_;

303

304  if (!is\_constant\_struct<T\_y>::value)

305  operands\_and\_partials.d\_x1[n] += rep\_deriv;

306  if (!is\_constant\_struct<T\_loc>::value)

307  operands\_and\_partials.d\_x2[n] -= rep\_deriv;

308  if (!is\_constant\_struct<T\_scale>::value)

309  operands\_and\_partials.d\_x3[n] -= rep\_deriv \* diff;

310  if (!is\_constant\_struct<T\_shape>::value)

311  operands\_and\_partials.d\_x4[n] += -2.0 \* exp(-0.5 \* diff\_sq \* (1.0

312  + alpha\_dbl\_sq)) / ((1 + alpha\_dbl\_sq) \* 2.0 \* pi()) / cdf\_log\_;

313  }

314

315  return operands\_and\_partials.to\_var(cdf\_log);

316  }

317

318  template <typename T\_y, typename T\_loc, typename T\_scale, typename T\_shape>

319  typename return\_type<T\_y,T\_loc,T\_scale,T\_shape>::type

320  skew\_normal\_ccdf\_log(const T\_y& y, const T\_loc& mu, const T\_scale& sigma,

321  const T\_shape& alpha) {

322  static const char\* function = "stan::prob::skew\_normal\_ccdf\_log(%1%)";

323

324  using stan::math::check\_positive;

325  using stan::math::check\_finite;

326  using stan::math::check\_not\_nan;

327  using stan::math::check\_consistent\_sizes;

328  using stan::math::owens\_t;

329  using stan::math::value\_of;

330

331  double ccdf\_log(0.0);

332

333  // check if any vectors are zero length

334  if (!(stan::length(y)

335  && stan::length(mu)

336  && stan::length(sigma)

337  && stan::length(alpha)))

338  return ccdf\_log;

339

340  check\_not\_nan(function, y, "Random variable", &ccdf\_log);

341  check\_finite(function, mu, "Location parameter", &ccdf\_log);

342  check\_not\_nan(function, sigma, "Scale parameter", &ccdf\_log);

343  check\_positive(function, sigma, "Scale parameter", &ccdf\_log);

344  check\_finite(function, alpha, "Shape parameter", &ccdf\_log);

345  check\_not\_nan(function, alpha, "Shape parameter", &ccdf\_log);

346  check\_consistent\_sizes(function,

347  y,mu,sigma,alpha,

348  "Random variable","Location parameter",

349  "Scale parameter","Shape paramter",

350  &ccdf\_log);

351

352  agrad::OperandsAndPartials<T\_y, T\_loc, T\_scale, T\_shape>

353  operands\_and\_partials(y, mu, sigma, alpha);

354

355  using stan::math::SQRT\_2;

356  using stan::math::pi;

357

358  VectorView<const T\_y> y\_vec(y);

359  VectorView<const T\_loc> mu\_vec(mu);

360  VectorView<const T\_scale> sigma\_vec(sigma);

361  VectorView<const T\_shape> alpha\_vec(alpha);

362  size\_t N = max\_size(y, mu, sigma, alpha);

363  const double SQRT\_TWO\_OVER\_PI = std::sqrt(2.0 / stan::math::pi());

364

365  for (size\_t n = 0; n < N; n++) {

366  const double y\_dbl = value\_of(y\_vec[n]);

367  const double mu\_dbl = value\_of(mu\_vec[n]);

368  const double sigma\_dbl = value\_of(sigma\_vec[n]);

369  const double alpha\_dbl = value\_of(alpha\_vec[n]);

370  const double alpha\_dbl\_sq = alpha\_dbl \* alpha\_dbl;

371  const double diff = (y\_dbl - mu\_dbl) / sigma\_dbl;

372  const double diff\_sq = diff \* diff;

373  const double scaled\_diff = diff / SQRT\_2;

374  const double scaled\_diff\_sq = diff\_sq \* 0.5;

375  const double ccdf\_log\_ = 1.0 - 0.5 \* erfc(-scaled\_diff) + 2 \* owens\_t(diff,

376  alpha\_dbl);

377  //ccdf\_log

378  ccdf\_log += log(ccdf\_log\_);

379

380  //gradients

381  const double deriv\_erfc = SQRT\_TWO\_OVER\_PI \* 0.5 \* exp(-scaled\_diff\_sq)

382  / sigma\_dbl;

383  const double deriv\_owens = erf(alpha\_dbl \* scaled\_diff)

384  \* exp(-scaled\_diff\_sq) / SQRT\_TWO\_OVER\_PI / (-2.0 \* pi()) / sigma\_dbl;

385  const double rep\_deriv = (-2.0 \* deriv\_owens + deriv\_erfc) / ccdf\_log\_;

386

387  if (!is\_constant\_struct<T\_y>::value)

388  operands\_and\_partials.d\_x1[n] -= rep\_deriv;

389  if (!is\_constant\_struct<T\_loc>::value)

390  operands\_and\_partials.d\_x2[n] += rep\_deriv;

391  if (!is\_constant\_struct<T\_scale>::value)

392  operands\_and\_partials.d\_x3[n] += rep\_deriv \* diff;

393  if (!is\_constant\_struct<T\_shape>::value)

394  operands\_and\_partials.d\_x4[n] -= -2.0 \* exp(-0.5 \* diff\_sq \* (1.0

395  + alpha\_dbl\_sq)) / ((1 + alpha\_dbl\_sq) \* 2.0 \* pi()) / ccdf\_log\_;

396  }

397

398  return operands\_and\_partials.to\_var(ccdf\_log);

399  }

400

401  template <class RNG>

402  inline double

403  skew\_normal\_rng(const double mu,

404  const double sigma,

405  const double alpha,

406  RNG& rng) {

407  boost::math::skew\_normal\_distribution<>dist (mu, sigma, alpha);

408

409  static const char\* function = "stan::prob::skew\_normal\_rng(%1%)";

410

411  using stan::math::check\_positive;

412  using stan::math::check\_finite;

413

414  check\_finite(function, mu, "Location parameter", (double\*)0);

415  check\_finite(function, alpha, "Shape parameter", (double\*)0);

416  check\_positive(function, sigma, "Scale parameter", (double\*)0);

417

418  return quantile(dist, stan::prob::uniform\_rng(0.0,1.0,rng));

419  }

420  }

421 }

422 #endif

423

traits.hpp

stan::agrad::erf

fvar< T > erf(const fvar< T > &x)

**Definition:** erf.hpp:17

stan::agrad::OperandsAndPartials::to\_var

T\_return\_type to\_var(double logp)

**Definition:** partials\_vari.hpp:138

uniform.hpp

error\_handling.hpp

stan::length

size\_t length(const T &)

**Definition:** traits.hpp:159

stan::agrad::erfc

fvar< T > erfc(const fvar< T > &x)

**Definition:** erfc.hpp:17

stan::DoubleVectorView

DoubleVectorView allocates double values to be used as intermediate values.

**Definition:** traits.hpp:358

stan::prob::skew\_normal\_cdf\_log

return\_type< T\_y, T\_loc, T\_scale, T\_shape >::type skew\_normal\_cdf\_log(const T\_y &y, const T\_loc &mu, const T\_scale &sigma, const T\_shape &alpha)

**Definition:** skew\_normal.hpp:237

stan::math::check\_finite

bool check\_finite(const char \*function, const T\_y &y, const char \*name, T\_result \*result)

Checks if the variable y is finite.

**Definition:** check\_finite.hpp:52

partials\_vari.hpp

stan::agrad::value\_of

T value\_of(const fvar< T > &v)

Return the value of the specified variable.

**Definition:** value\_of.hpp:16

constants.hpp

stan::is\_vector

**Definition:** traits.hpp:83

stan::agrad::OperandsAndPartials

A variable implementation that stores operands and derivatives with respect to the variable...

**Definition:** partials\_vari.hpp:76

traits.hpp

stan::return\_type::type

boost::math::tools::promote\_args< typename scalar\_type< T1 >::type, typename scalar\_type< T2 >::type, typename scalar\_type< T3 >::type, typename scalar\_type< T4 >::type, typename scalar\_type< T5 >::type, typename scalar\_type< T6 >::type >::type type

**Definition:** traits.hpp:406

stan::prob::skew\_normal\_cdf

return\_type< T\_y, T\_loc, T\_scale, T\_shape >::type skew\_normal\_cdf(const T\_y &y, const T\_loc &mu, const T\_scale &sigma, const T\_shape &alpha)

**Definition:** skew\_normal.hpp:141

stan::math::owens\_t

boost::math::tools::promote\_args< T1, T2 >::type owens\_t(const T1 &h, const T2 &a)

The Owen's T function of h and a.

**Definition:** owens\_t.hpp:64

stan::is\_constant\_struct

Metaprogram to determine if a type has a base scalar type that can be assigned to type double...

**Definition:** traits.hpp:57

stan::math::value\_of

double value\_of(const T x)

Return the value of the specified scalar argument converted to a double value.

**Definition:** value\_of.hpp:24

stan::prob::include\_summand

Template metaprogram to calculate whether a summand needs to be included in a proportional (log) prob...

**Definition:** traits.hpp:35

stan::prob::skew\_normal\_rng

double skew\_normal\_rng(const double mu, const double sigma, const double alpha, RNG &rng)

**Definition:** skew\_normal.hpp:403

stan::math::SQRT\_2

const double SQRT\_2

The value of the square root of 2, .

**Definition:** constants.hpp:20

stan::agrad::owens\_t

fvar< T > owens\_t(const fvar< T > &x1, const fvar< T > &x2)

**Definition:** owens\_t.hpp:17

stan::agrad::sqrt

fvar< T > sqrt(const fvar< T > &x)

**Definition:** sqrt.hpp:15

stan::agrad::OperandsAndPartials::d\_x2

VectorView< double \*, is\_vector< T2 >::value, is\_constant\_struct< T2 >::value > d\_x2

**Definition:** partials\_vari.hpp:83

stan::prob::skew\_normal\_log

return\_type< T\_y, T\_loc, T\_scale, T\_shape >::type skew\_normal\_log(const T\_y &y, const T\_loc &mu, const T\_scale &sigma, const T\_shape &alpha)

**Definition:** skew\_normal.hpp:23

stan::prob::uniform\_rng

double uniform\_rng(const double alpha, const double beta, RNG &rng)

**Definition:** uniform.hpp:339

stan::math::check\_consistent\_sizes

bool check\_consistent\_sizes(const char \*function, const T1 &x1, const T2 &x2, const char \*name1, const char \*name2, T\_result \*result)

**Definition:** check\_consistent\_sizes.hpp:13

stan::max\_size

size\_t max\_size(const T1 &x1, const T2 &x2)

**Definition:** traits.hpp:191

stan::math::check\_positive

bool check\_positive(const char \*function, const T\_y &y, const char \*name, T\_result \*result)

**Definition:** check\_positive.hpp:54

owens\_t.hpp

stan::prob::skew\_normal\_ccdf\_log

return\_type< T\_y, T\_loc, T\_scale, T\_shape >::type skew\_normal\_ccdf\_log(const T\_y &y, const T\_loc &mu, const T\_scale &sigma, const T\_shape &alpha)

**Definition:** skew\_normal.hpp:320

stan::math::check\_not\_nan

bool check\_not\_nan(const char \*function, const T\_y &y, const char \*name, T\_result \*result)

Checks if the variable y is nan.

**Definition:** check\_not\_nan.hpp:56

stan::agrad::OperandsAndPartials::d\_x4

VectorView< double \*, is\_vector< T4 >::value, is\_constant\_struct< T4 >::value > d\_x4

**Definition:** partials\_vari.hpp:85

value\_of.hpp

stan::agrad::OperandsAndPartials::d\_x1

VectorView< double \*, is\_vector< T1 >::value, is\_constant\_struct< T1 >::value > d\_x1

**Definition:** partials\_vari.hpp:82

stan::agrad::OperandsAndPartials::d\_x3

VectorView< double \*, is\_vector< T3 >::value, is\_constant\_struct< T3 >::value > d\_x3

**Definition:** partials\_vari.hpp:84

stan::math::pi

double pi()

Return the value of pi.

**Definition:** constants.hpp:77

stan::agrad::log

fvar< T > log(const fvar< T > &x)

**Definition:** log.hpp:15

stan::VectorView

VectorView is a template metaprogram that takes its argument and allows it to be used like a vector...

**Definition:** traits.hpp:275

stan::agrad::exp

fvar< T > exp(const fvar< T > &x)

**Definition:** exp.hpp:16

stan::math::dist

double dist(const std::vector< double > &x, const std::vector< double > &y)

**Definition:** dist.hpp:11


---

[ Stan Home Page ]

*© 2011–2014,
Stan Development Team.*
